# Slow‐replicating leukemia cells represent a leukemia stem cell population with high cell‐surface CD74 expression

**DOI:** 10.1002/1878-0261.13690

**Published:** 2024-06-22

**Authors:** Huan Li, Zhijie Cao, Yiming Liu, Zhenya Xue, Yishuang Li, Haiyan Xing, Yingxi Xu, Runxia Gu, Shaowei Qiu, Hui Wei, Min Wang, Qing Rao, Jianxiang Wang

**Affiliations:** ^1^ State Key Laboratory of Experimental Hematology, National Clinical Research Center for Blood Diseases, Haihe Laboratory of Cell Ecosystem, Tianjin Key Laboratory of Cell Therapy for Blood Diseases, Institute of Hematology & Blood Diseases Hospital Chinese Academy of Medical Sciences & Peking Union Medical College Tianjin China

**Keywords:** acute myeloid leukemia, CD74, chemotherapy resistance, leukemia stem cell, quiescence, self‐renewal

## Abstract

Persistence of quiescent leukemia stem cells (LSCs) after treatment most likely contributes to chemotherapy resistance and poor prognosis of leukemia patients. Identification of this quiescent cell population would facilitate eradicating LSCs. Here, using a cell‐tracing PKH26 (PKH) dye that can be equally distributed to daughter cells following cell division *in vivo*, we identify a label‐retaining slow‐cycling leukemia cell population from AML1‐ETO9a (AE9a) leukemic mice. We find that, compared with cells not maintaining PKH‐staining, a higher proportion of PKH‐retaining cells are in G0 phase, and PKH‐retaining cells exhibit increased colony formation ability and leukemia initiation potential. In addition, PKH‐retaining cells possess high chemo‐resistance and are more likely to be localized to the endosteal bone marrow region. Based on the transcriptional signature, HLA class II histocompatibility antigen gamma chain (*Cd74*) is highly expressed in PKH‐retaining leukemia cells. Furthermore, cell surface CD74 was identified to be highly expressed in LSCs of AE9a mice and CD34^+^ human leukemia cells. Compared to Lin^−^CD74^−^ leukemia cells, Lin^−^CD74^+^ leukemia cells of AE9a mice exhibit higher stemness properties. Collectively, our findings reveal that the identified slow‐cycling leukemia cell population represents an LSC population, and CD74^+^ leukemia cells possess stemness properties, suggesting that CD74 is a candidate LSC surface marker.

## Graphical abstract

Using a cell‐tracing PKH dye in AML1‐ETO9a leukemic mice, we identified a label‐retaining leukemia cell population that maintained quiescence, had self‐renewal potential and was chemo‐resistant. PKH‐retaining and endosteum‐localized leukemia cells had high *Cd74* expression. Cell surface CD74 was highly expressed in leukemia stem cells of leukemic mice and AML patients. CD74^+^ leukemia cells exhibit stemness properties.

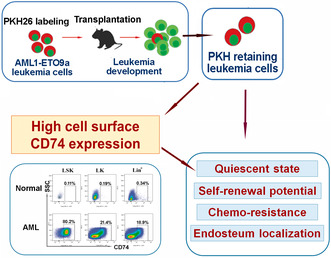

**Abbreviations**
AE9a: AML1‐ETO9a
AML: acute myeloid leukemia
BM: bone marrow
CBM: central bone marrow
EBM: endosteal surface of bone marrow
FACS: Fluorescence‐activated cell sorting.
HSCs: hematopoietic stem cells
LK: Lin^−^c‐Kit^+^
LSCs: leukemia stem cells
LSK: Lin^−^c‐Kit^+^Sca‐1^+^
MFI: mean fluorescence intensity

## 1 Introduction

Leukemia stem cells (LSCs) population is present in a small proportion of leukemia cells, which defined by their capacity to self‐renew extensively, give rise to heterogeneous leukemic cells, and initiate acute myeloid leukemia (AML) in immune‐deficient mice [1, 2, 3]. Moreover, LSCs are cell cycle quiescent and chemotherapy resistant, which may be facilitated by LSCs to escape the killing effect of chemotherapy and be attributed to disease relapse [4].

Quiescence is one of the key properties of LSC and is shared with hematopoietic stem cells (HSC) [5]. For LSC, quiescence and dormancy are not only importance for long‐term self‐renewal potential, but also responsible for resistance to conventional chemotherapy [4, 6]. Although several intrinsic and extrinsic regulators of quiescence in normal HSC have been well defined [7, 8, 9], the difference in quiescence regulation between LSC and HSC is less well understood. Since quiescent LSCs are directly linked to chemotherapy resistance and the poor prognosis of leukemia, it is important to define this population and understand the mechanisms that govern LSC's quiescence. In addition to this, identification of LSC‐specific cell surface marker for targeting quiescent LSCs will facilitate the development of new therapeutic strategies to eradicating LSCs [10].

As a fluorescent dye, PKH has been used to trace the migration of stem cells and carcinoma cells [11, 12]. Due to its stability *in vivo* and *in vitro*, following labeling, the fluorescence can persist for a long time [13]. Upon each cell division, the PKH labeling could be equally distributed to the daughter cells. For PKH‐labeled leukemia cells, maintaining fluorescence density in the cells means that these cells keep slow‐cycling and may represent the quiescent LSCs. In the present study, using PKH26 labeling on the leukemia cells from AML1‐ETO9a mouse leukemia model (AE9a), we identified that the fluorescence retention cells represented LSC population in quiescence state, and PKH26‐positive cells possessed properties of LSCs, including self‐renewal, chemotherapy resistance, and leukemia initiation potential. Furthermore, to investigate possible targets for eradicating LSCs, based on the transcriptional patterns of PKH26‐positive cells and identified leukemia cells with stemness properties, we found that *Cd74* was highly expressed in both PKH^+^ and endosteal‐resident leukemia cells.

As an MHC class II chaperone, CD74 was originally identified to play a role in antigen presentation. In addition to its cytoplasmic form in the MHC II antigen presentation pathway, CD74 is also a plasma membrane protein [14]. Corresponding to its function in MHC II antigen presentation, CD74 is mainly expressed in dendritic cells, B cells, and macrophages in normal tissue [14, 15, 16]. As a cell surface receptor of macrophage migration inhibitory factor (MIF), CD74 has been identified to be expressed in a variety of tumors, including multiple myeloma, breast cancer, gastric carcinoma [17, 18, 19]. In our further study, cell surface CD74 expression in mouse leukemia cells with stemness properties was identified. The expression of CD74 in human AML LSCs and the association of CD74 positive leukemia cells with stemness properties in leukemia mice were also validated.

## 2 Materials and methods

### 2.1 Human samples

Human samples were obtained from AML patients and healthy donors enrolled at the Blood Diseases Hospital, the Chinese Academy of Medical Sciences. All participants included in the study signed informed consents in accordance with the Declaration of Helsinki. The study methodologies also conformed to the standards set by the Declaration of Helsinki, and were performed in accordance with the ethical standards approved by the Institutional Review Board of Institute of Hematology and Blood Diseases Hospital (Ref: NKRDP2021005‐EC‐2). The study samples were collected between January 2022 and December 2023.

### 2.2 Animals and mouse AML transplantable model

All experiments involving mice were approved by the Institutional Animal Care and Use Committees of the State Key Laboratory of Experimental Hematology (License number: HCAMS‐DWLL‐NSFC2023115‐1). 6–8‐week‐old female C57BL/6 mice were purchased from Beijing Vital River Laboratory Animal Technology Co., Ltd (Beijing, China) and used for all animal experiments. The mice were kept in a specific pathogen free unit with standard laboratory chow and drinking water. The AML1‐ETO9a (AE9a) fusion‐gene‐induced mouse AML transplantable model was previously established in our laboratory, which were originally obtained from 6 to 8‐week‐old female C57BL/6 mice [20]. The recipient mice developed leukemia with an average latency of 25 days following being transplanted with 10^6^ AE9a leukemia cells. Green fluorescent protein (GFP^+^) cells in the recipient represent AE9a leukemia cells.

### 2.3 PKH26 labeling

GFP^+^ cells from AE9a leukemia mice were sorted by FACS and then stained with red PKH26 dye according to the instruction of PKH26 Fluorescent Cell Linker Kit (Sigma Aldrich). Briefly, 10^7^ cells were suspended in 1.0 mL of Diluent C, and stained by admixing rapidly with 1.0 mL of PKH26 working solution (8 μL PKH26 dye solution in 1.0 mL of Diluent C) for 5 min. Final staining concentration was 4 μm PKH26. Staining was stopped by addition of 1 mL of serum and the cells were then washed with culture medium with serum.

### 2.4 Cells at G0 phase of cell cycle assay

Cells were stained with PE‐Ki67 and 7‐aminoactinomycin D (7‐AAD) or DAPI as described previously [21]. The ratio of cells at G0 phase was detected and analyzed by Flow cytometry (LSRII flow cytometer, BD Biosciences, San Jose, CA, USA).

### 2.5 Colony formation assay

10^4^ leukemia cells were plated in 48‐well or 24‐well plates in triplicates in MethoCult M3434 (STEMCELL Technologies Inc., Vancouver, BC, Canada). Colonies consisting of more than 40 cells were counted 7 days later. Secondary colony‐forming units assay was performed by harvesting the cells from the primary cultures and re‐plating the cells in same number of 10^4^ cells per well into new plates. Tertiary assay was performed in the same way.

### 2.6 Isolation of endosteal bone marrow leukemia cells

AE9a leukemic mice used for BM isolation were obtained from 6 to 8‐week‐old female C57BL/6 mice transplanted with AE9a leukemia cells. Isolations of central and endosteal bone marrow cells were performed as described [7, 22]. Briefly, central marrow cells were obtained by flushing the femurs and tibia of AE9a mice, the flushed cells represent central bone marrow (CBM) cells. After BM cells were flushed out, femurs and tibias were cut into small pieces. After being treated with 2 mg/mL collagenase I (Sigma) in a shaking incubator at 37 °C for 15 min, the bone fragments were then washed and filtered through 70 μm cell strainer, and the cells were collected, which represent endosteal bone marrow (EBM) cells. BM cells represent total bone marrow leukemia cells collected from the supernatant of crushed femurs and tibias. GFP^+^ leukemia cells were sorted or analyzed by FACS for further analysis.

### 2.7 Microarray and RNA‐seq analysis

For the Affymetrix microarray assay on PKH^+/−^ cells, the mRNA expression profiling was determined using the murine Clariom S Array (Affymetrix GeneChip®, Santa Clara, CA, USA). The data were analyzed with Robust Multichip Analysis (RMA) algorithm using the Affymetrix default analysis settings and global scaling as a normalization method.

For RNA‐Seq assay on CBM and EBM cells, the sequencing libraries were constructed using the rRNA‐depleted RNA by NEBNext® Ultra™ Directional RNA Library Prep Kit for Illumina® (NEB, Ipswich, MA, USA). The quality of RNA‐seq libraries was evaluated on the Agilent Bioanalyzer 2100 system. After cluster generation, the libraries were sequenced on an Illumina Hiseq 4000 platform and 150 bp paired‐end reads were generated.

### 2.8 Statistical analysis

Comparisons between two groups were determined using Student's *t*‐test and *P* values < 0.05 were considered statistically significant differences. Data of triplicate experiments were shown as mean value ± SEM.

## 3 Results

### 3.1 PKH26 labeled retention cells from AE9a leukemia mouse are more quiescent

**Fig. 1:**
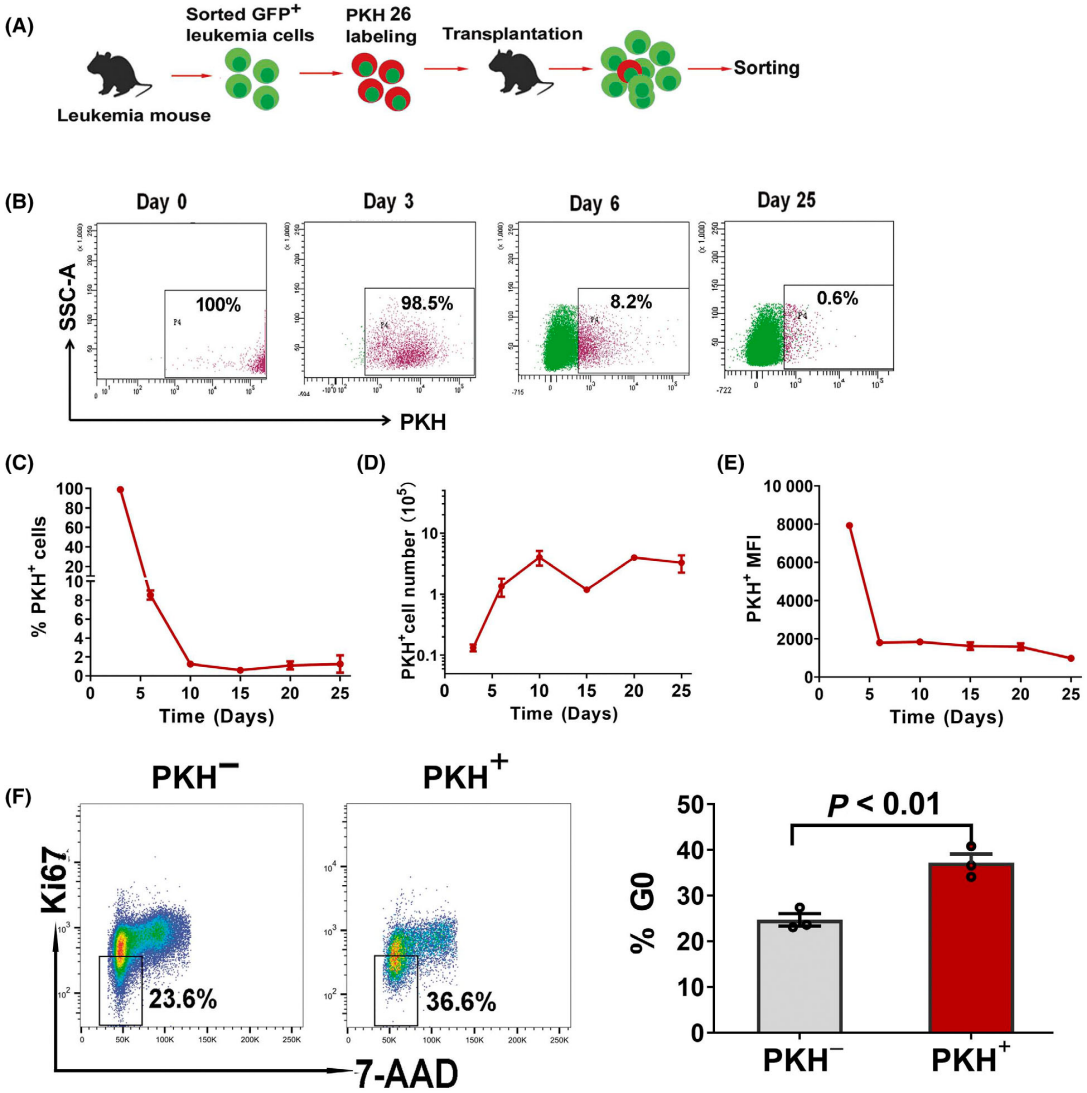
High percentage of cells in G0 phase of the cell cycle within PKH26 retention leukemia cell population from AE9a leukemia mouse. (A) Experimental schema for PKH26 staining and analysis on PKH26 retention cells from the mice transplanted with PKH26‐stained leukemia cells. (B) Representative flow cytometry plots on PKH intensity profiles of GFP^+^ cells from PKH26‐stained leukemia cells transplanted mice at day 0, 3, 6 and 25. (C–E) Monitoring of kinetics of PKH‐positive cell number and PKH intensity in bone marrow of the mice after PKH26‐stained leukemia cells *transplantation*. The data at each time point are presented as the mean ± SD from two mice. (F) G0 phase of the cell cycle analyzed by flow cytometry. PKH^+^ and PKH^−^ cells were sorted from bone marrow of the mice transplanted with PKH26‐labeled leukemia cells on day 25 after transplantation, and then subjected to G0 phase analysis. One representative flow cytometry plot for G0 phase analysis is shown in the left panel. The graph on the right panel is the percentage of cells in G0 phase. Data are presented as the mean ± SEM from three independent experiments. Each dot represents the value from one experiment. The statistical significance were determined using unpaired *t*‐test.

Firstly, we monitored the fluorescence intensity of PKH26 labeled leukemia cells after transplanting them into recipient mice. Fluorescence‐activated cell sorting (FACS) sorted GFP^+^ leukemia cells were obtained from AE9a leukemia mouse. Following PKH26 staining *in vitro*, PKH26‐stained cells were transplanted into sublethally irradiated C57BL/6 recipient mice (Fig. 1A). Fluorescence intensity of PKH in GFP^+^ leukemia cells from BM of recipient mice were monitored. The proliferation of GFP^+^ leukemia cells in BM of recipient mice is shown in Fig. S1. After the PKH26‐stained leukemia cells were transplanted and proliferated *in vivo*, a decrease of PKH fluorescence intensity in GFP^+^ leukemia cells could be observed. As shown in Fig. 1B,C, the percentage of PKH‐positive cells was up to 100% following staining, after transplantation, the percentage of PKH‐positive cells decreased gradually with *in vivo* cell division. Figure 1D,E showed that the number of PKH‐positive cells increased from day 1 to day 10 and then maintained at a stable level *in vivo*, however, the fluorescence intensity of PKH‐positive cells was decreased and kept at a lower level. With the division of PKH high cells into PKH intermediate and PKH low cells, and increasing of PKH‐negative cells, the number of PKH low cells increased and the fluorescence intensity and the percentage of PKH‐positive cells decreased. This indicated that following the division of leukemia cells and the segregation of PKH26 into daughter cells, a very small population of slowly proliferating cells retained relatively low dye loading. Until 25 days after transplantation, the mice develop leukemia and the percentage of PKH retention cells remained at a very low level (Fig. 1C). These PKH retention cells were supposed to be the cells with slow cell cycling and quiescent.

To identify that PKH maintaining cells are a small population of quiescent cells, we sorted PKH‐positive cells (PKH^+^) and PKH‐negative cells (PKH^−^) according to the fluorescence intensity from BM of the mouse transplanted with PKH26‐labeled leukemia cells on day 25 after transplantation. The percentage of cells in G0 phase in each sorted population was then determined based on 7‐AAD and Ki‐67 staining. As shown in Fig. 1F, comparing to PKH^−^ cells, there was a higher proportion of PKH^+^ leukemia cells maintaining in G0 phase (*P* < 0.01), indicating that PKH‐positive cells have a higher proportion of cells in quiescent state.

### 3.2 PKH26‐labeled retention cells exhibit increased colony formation ability and leukemia initiation potential

**Fig. 2:**
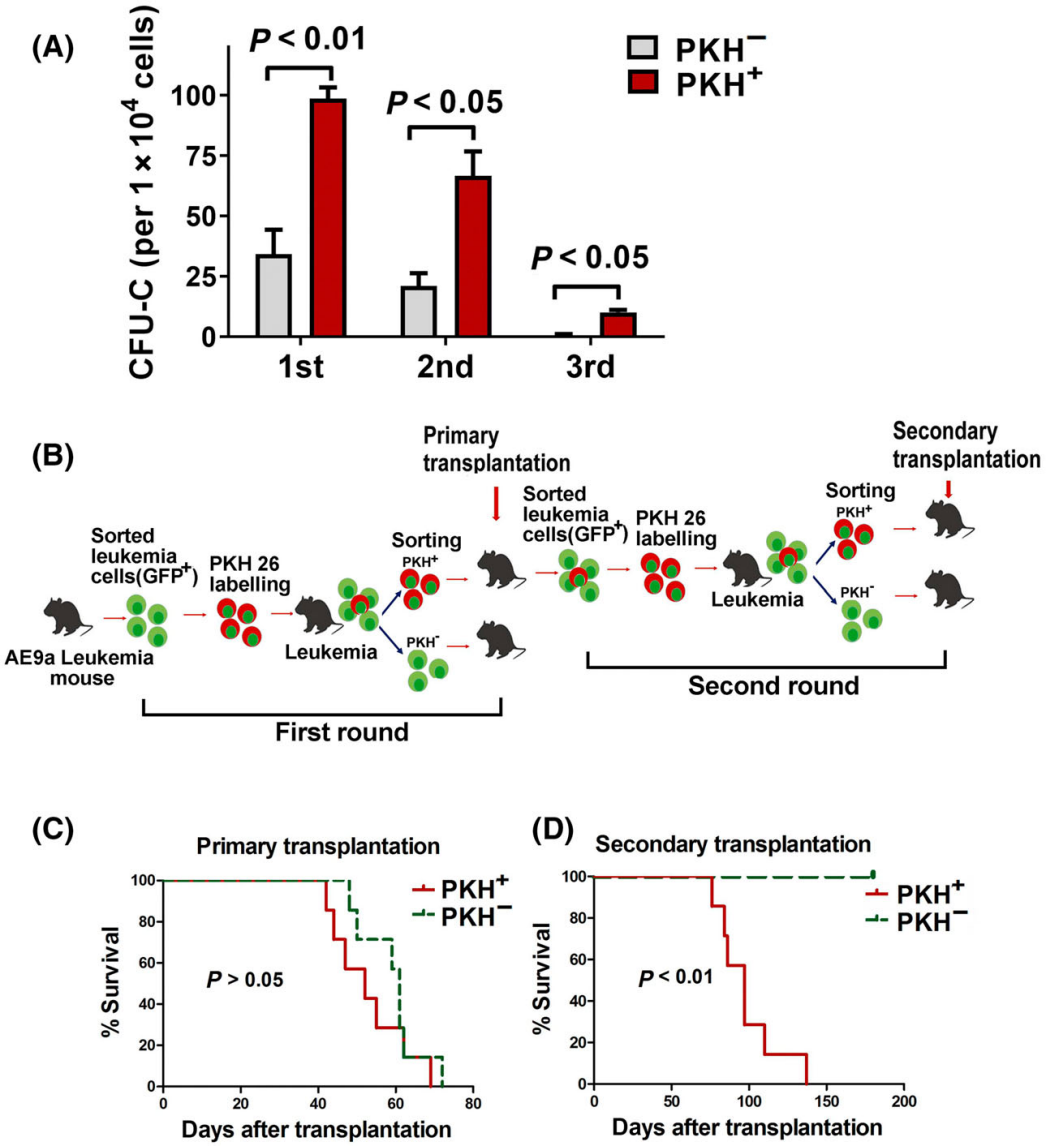
Enhanced colony formation ability and leukemia initiation potential of PKH26 retention leukemia cells. (A) Colony formation ability of PKH^+^ and PKH^−^ leukemia cells. PKH^+^ and PKH^−^ cells were sorted from GFP^+^ bone marrow cells of PKH26‐stained leukemia cells transplanted mice on day 25 after transplantation and colony formation assay was then performed. Data are presented as the mean ± SEM of three independent experiments. The statistical significance was determined using unpaired *t*‐test. (B) Experimental schema for PKH26 staining and the serial transplantation assay. Equal number of sorted PKH^+^ or PKH^−^ cells was used in both the first round and the second round of transplantation. (C) Kaplan–Meier survival curves of primary recipients inoculated with PKH^+^ or PKH^−^ leukemia cells (PKH^+^: *n* = 7, PKH^−^: *n* = 7). The statistical significance of survival was determined using Log‐rank (Mantel–Cox) test. (D) Kaplan–Meier survival curves of the second transplantation (PKH^+^: *n* = 7, PKH^−^: *n* = 5). Transplanted PKH^+^ or PKH^−^ leukemia cells were sorted from secondary round recipients. For the second round of transplantation, GFP^+^ cells from primary leukemia mice transplanted with PKH^+^ cells were sorted and re‐stained with PKH26, and then injected into sublethally irradiated recipients. When these recipients developed leukemia, PKH^+^ and PKH^−^ cells were sorted and second transplantation was performed. The statistical significance of survival was determined using Log‐rank (Mantel–Cox) test.

Quiescence of LSCs always goes along with their potential for long‐term self‐renewal. To test whether the slow‐replicating property may be the response to increased self‐renewal, the ability of colony formation of PKH^+^ and PKH^−^ cells sorted from PKH26‐labeled leukemia cells transplanted mice was then determined by serial colony formation assay. Figure 2A showed that in the first round plating, compared to PKH^−^ cells, PKH^+^ cells from BM of mice transplanted with PKH26‐labeled leukemia cells produced more colonies. The number of colonies derived from PKH^+^ cells was significantly higher than that from PKH^−^ cells (*P* < 0.01). In the second and third round plating, PKH^+^ cells also produced significantly more colonies (*P* < 0.05). This implies that PKH^+^ slow‐replicating leukemia cells display higher self‐renewal potential.

To further evaluate self‐renewal and repopulating ability of slow‐replicating cells, we then performed serial transplantation assays. A total of 1000 sorted PKH^+^ and PKH^−^ cells were used in serial transplantation assay respectively. The experimental strategy for serial transplantation is shown in Fig. 2B. In the primary recipients that transplanted with PKH^+^ and PKH^−^ cells, all mice developed AML with similar survival time. There was no significant difference in Kaplan–Meier survival between PKH^+^ and PKH^−^ cell groups (Fig. 2C). Then, the second round of transplantation was performed. GFP^+^ leukemia cells from the first recipients transplanted with PKH^+^ cells were then re‐stained with PKH26 and re‐transplanted into recipient mice. After 25 days of transplantation, PKH^+^ and PKH^−^ cells were sorted and transplanted into recipients (secondary transplantation). Figure 2D showed that in the secondary transplantation, 100% of PKH^+^ cell transplanted mice developed leukemia, however, the recipients transplanted with PKH^−^ cells did not develop leukemia until 180 days post‐transplantation. The survival time of mice in PKH^−^ cell group was significantly longer than that in PKH^+^ cell group. These results thus suggest that PKH^−^ cells with a low frequency of cells in quiescent state are more likely to be exhausted and eventually lose their leukemia initiation capacity, whereas PKH^+^ cells, representing slow‐replicating LSC, are capable of long‐term leukemia initiation potential.

### 3.3 PKH‐positive leukemia cells exhibit chemotherapy resistance and are more likely to be localized to the EBM

**Fig. 3:**
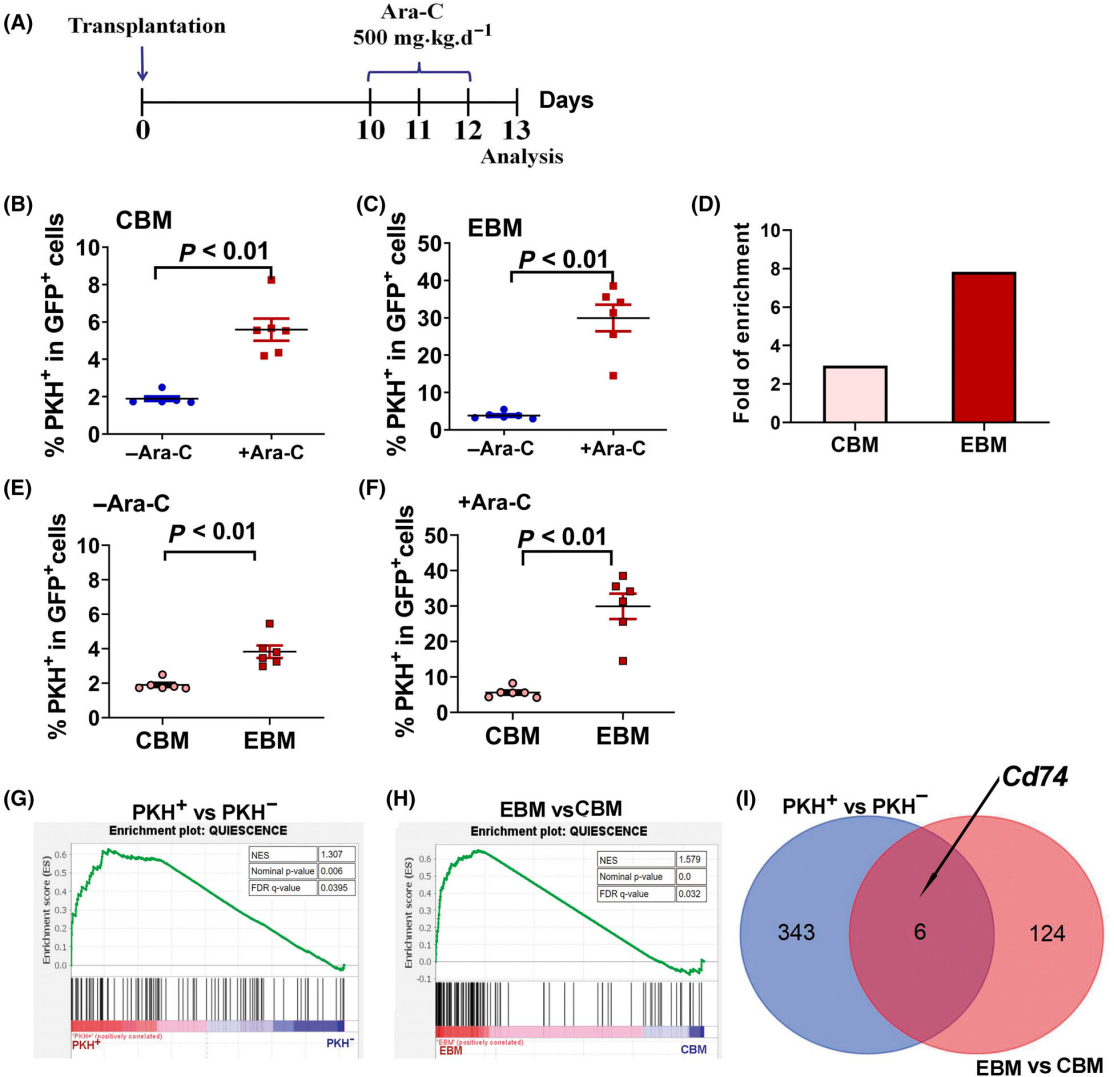
Enrichment of PKH^+^ leukemia cell population by chemotherapy *in vivo*. (A) Ara‐C treatment regimen *in vivo*. The mice received 500 mg/kg Ara‐C for 3 days beginning on day 10 after PKH26‐stained leukemia cells transplantation. PKH^+^ cell population were evaluated 1 day after the last Ara‐C treatment. (B) The ratio of PKH^+^/ GFP^+^ cells from flushed bone marrow (CBM) of PKH26‐stained leukemia cells transplanted mice treated with or without Ara‐C. (C) The ratio of PKH^+^/ GFP^+^ cells from collagenase digested fragments (EBM) of PKH26‐stained leukemia cells transplanted mice treated with or without Ara‐C. (D) The fold of enrichment by Ara‐C treatment in CBM and EBM. Data are obtained by dividing the average value of PKH^+^/GFP^+^% from six Ara‐C‐treated mice by that value from six Ara‐C‐untreated mice. (E) Percentage of PKH^+^ cells in GFP^+^ cells of CBM and EBM from untreated mice. (F) Percentage of PKH^+^ cells in GFP^+^ cells of CBM and EBM from Ara‐C‐treated mice. The data are presented as the mean ± SEM from six mice and the statistical significances were determined using unpaired t‐test (B, C, E, F). (G) Enrichment plot from the gene set enrichment analysis (GSEA) for quiescence‐related genes in PKH^+^ cell population. (H) Enrichment plot from GSEA for quiescence‐related genes in EBM leukemia cells group. (I) Venn analysis of significantly upregulated genes in PKH^+^ vs. PKH^−^ group and EBM vs. CBM group. CBM, central bone marrow; EBM, endosteal surface of bone marrow.

Quiescence is essential for chemotherapy resistance of LSCs, we then asked whether PKH^+^ slow‐replicating leukemia cells were resistant to chemotherapy drug and could be enriched after Ara‐C treatment. To address this, we performed a chemotherapy enrichment assay. The experimental strategy is shown in Fig. 3A. On day 10 after PKH‐labeled AE9a leukemia cells were transplanted into mice, the recipients received 500 mg/kg Ara‐C for 3 days and then PKH^+^ cell population was determined 1 day after the last Ara‐C treatment. Figure 3B showed that in flushed BM (CBM) of Ara‐C‐treated mice, the percentage of PKH^+^ cell population was significantly higher than that in untreated mice, PKH^+^ cells were enriched 2.95 folds by Ara‐C treatment. These data showed that PKH^+^ cells exhibited high drug resistance, indicating that these slow‐replicating cells are more resistant to chemotherapy drug.

Several investigations demonstrate that bone marrow, especially osteoblast‐rich area is an important LSCs engraft niche, where LSCs are quiescent and protected from chemotherapy [23]. We then explored whether PKH^+^ quiescence cells localized to the endosteal surface of BM specifically exhibited the most chemotherapy resistance. We then compared the percentage of PKH^+^ cell population in the GFP^+^ leukemia cells from CBM and collagenase‐treated bone of Ara‐C‐treated mice. The leukemia cells that tightly adhere to bone were obtained by collagenase digestion of the bone after BM cells were flushed out, which was defined as the cells located in endosteal surface of BM (EBM). As shown in Fig. 3C, after Ara‐C treatment, the percentage of PKH^+^ cell population in EBM was 29.94% (mean value), which was significantly higher than that from Ara‐C untreated mice (mean value, 3.825%) (*P* < 0.01). After Ara‐C treatment, the PKH^+^ cell population was engrafted 2.95‐fold in CBM, whereas it was engrafted 7.83‐fold in EBM. The enrichment of PKH^+^ cells by Ara‐C treatment in EBM was higher than that in CBM (Fig. 3D). In addition, when we compared PKH^+^ populations in EBM GFP^+^ cells with that in CBM of Ara‐C untreated mice, it was observed that percentages of PKH^+^ cell population in EBM were significantly higher than that in CBM (Fig. 3E), this demonstrating that PKH label retention cells are enriched in EBM. Similarly, in Ara‐C‐treated mice, the percentage of PKH^+^ cells in EBM GFP^+^ cells was also significantly higher than that in CBM GFP^+^ cells (Fig. 3F). Taken together, above results demonstrate that a significant proportion of chemo‐resistant PKH^+^ cells are more likely to be localized to the endosteal surface of BM and leukemia cells located in this area are more resistant to chemotherapy drug.

### 3.4 PKH‐positive leukemia cells exhibit a transcriptional signature enriched in quiescence and have a high CD74 expression

We then isolated PKH^+^ and PKH^−^ cell populations and performed transcriptome microarray assay. PKH^+^ and PKH^−^ leukemia cells displayed different transcriptional patterns. As expected, Gene Set Enrichment Analysis (GSEA) showed that the enrichment of the upregulated genes in PKH^+^ cell population fell into the quiescence gene set (Fig. 3G). Given that chemo‐resistant PKH^+^ cells are more likely to be localized to EBM, we also examined the transcriptional patterns of leukemia cells isolated from EBM and CBM using RNA‐seq analysis. Top genes upregulated in leukemia cell of PKH^+^ vs PKH^−^ and leukemia cells resided in EBM vs CBM were shown in Table S1. Consistent to PKH^+^ cell population, EBM leukemia cells also exhibit quiescence transcriptional signature (Fig. 3H). Differentially expressed genes (DEGs) were further identified between PKH^+^ vs. PKH^−^ leukemia cells and EBM vs. CBM, respectively. Figure 3I showed that six overlapping genes were obtained from the Venn analysis of the upregulated DEGs shared by these two simple pairs. *Cd74* was one of the overlapping genes, indicating that *Cd74* was highly expressed in both PKH^+^ and EBM leukemia cells when compared to their PKH^−^ and CBM counterparts. This suggests that CD74 is highly expressed in slow‐replicating cell population.

**Fig. 4:**
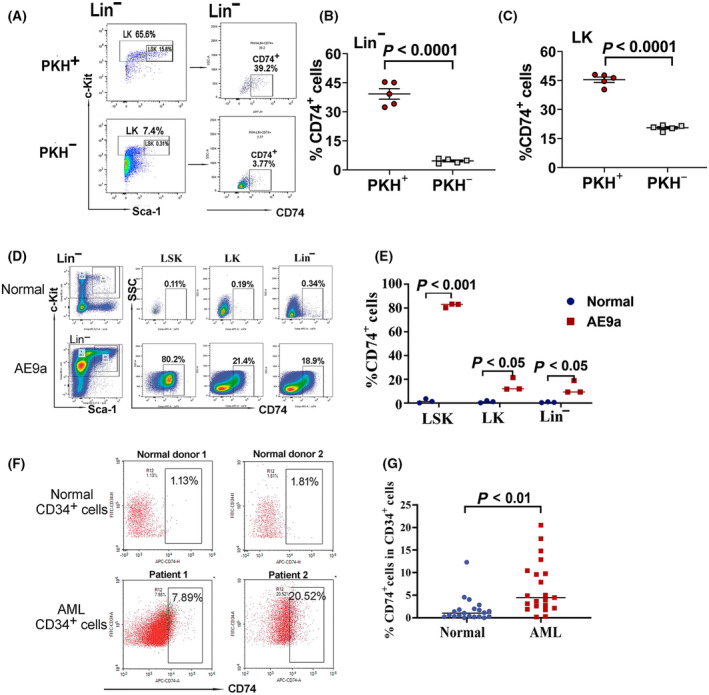
Cell surface CD74 expression in bone marrow of AE9a leukemia mouse and human AML leukemia stem cells. (A–C) Cell surface CD74 expression in PKH^+^ and PKH^−^ cell populations in bone marrow of PKH26‐stained leukemia cells transplanted mice. The ratio of CD74^+^ cells in PKH^+^ and PKH^−^ cell population was analyzed on day 10 after the mice being transplanted with PKH26‐stained leukemia cells. (A) Representative flow cytometry plots for cell surface CD74 expressions in Lin^−^ leukemia cells in PKH^+^ and PKH^−^ cell populations. (B) The percentage of CD74^+^ cells in PKH^+^ and PKH^−^ cell populations in Lin^−^ leukemia cells. (C) The percentage of CD74^+^ cells in PKH^+^ and PKH^−^ cell populations in Lin^−^c‐Kit^+^ (LK) leukemia cells. The data are presented as the mean ± SEM from five mice (B, C). (D) Representative flow cytometry plots for cell surface CD74 expressions in LSK (Lin^−^c‐Kit^+^Sca‐1^+^), LK (Lin^−^c‐Kit^+^) and Lin^−^ cell populations in normal and AE9a leukemia mice. (E) The percentage of CD74^+^ cells in LSK (Lin^−^c‐Kit^+^Sca‐1^+^), LK (Lin^−^c‐Kit^+^) and Lin^−^ cell populations in normal and AE9a leukemia mice. Data are presented as the mean ± SEM from three mice in each group. (F) Representative flow cytometry plot showing the expression of CD74 in CD34^+^ leukemia cells in normal bone marrow (from two normal donors) and AML patients (from two AML patients). (G) The ratio of CD74^+^/CD34^+^ cells in bone marrow of normal donors (*n* = 21) and AML patients (*n* = 21). The statistical significances were determined using unpaired *t*‐test (B, C, E, G).

CD74 can be expressed in the form of a cell surface protein, therefore, to confirm CD74 is highly expressed in slow‐replicating cell population and further identify cell surface CD74 expressing cells represent a cell population with stemness, we first detected cell surface CD74 in PKH^+^ and PKH^−^ AE9a leukemia cells from the mice transplanted with PKH26‐labeled leukemia cells. In Lin^−^GFP^+^ leukemia cells, the ratio of CD74^+^ cells in PKH^+^ and PKH^−^ cell population was analyzed. As shown in Fig. 4A,B, the mean value of the ratio of CD74^+^ in PKH^+^ cell population was 39.2%, which was significantly higher than that in PKH^−^ cell population (mean value: 4.6%). Similar result was also observed in Lin^−^c‐Kit^+^ (LK) cells (Fig. 4C). This indicates that cell surface CD74 is highly expressed in slow‐replicating cells and suggests that CD74^+^ leukemia cells may represent LSCs.

### 3.5 Cell surface CD74 is highly expressed in AE9a mouse myeloid leukemia cells and human acute myeloid leukemia stem cells

Above results indicate that CD74 was highly expressed in PKH^+^ quiescent leukemia cells population. We then focused on verifying whether CD74 is involved in maintaining the stemness properties of leukemia cells. The cell surface CD74 expressions in different cell population in BM of AE9a and normal mice were then determined. For BM cells in normal mice, cell surface CD74 was almost undetectable in Lin^−^ c‐Kit^+^ Sca‐1^+^ (LSK), LK or Lin^−^ cell population (Fig. 4D). However, in AE9a leukemia mice, cell surface CD74 was highly expressed in LSK leukemia cells (Fig. 4D). The percentage of CD74^+^ cell was significantly higher in LSK, LK and Lin^−^ cells of AE9a leukemia mice than that in the normal counterparts (Fig. 4E). The highest percentage of CD74^+^ cell was found in LSK leukemic cell population, which was 48‐fold higher compared to that in normal LSK cells. This result suggests that CD74 is highly expressed in LSCs of AE9a leukemia cells.

We then examined CD74 expression in BM CD34^+^ cells of normal donors and AML patients. CD34 positive leukemia samples were selected. AML CD34^+^ leukemia cells exhibited higher levels of CD74 expression compared with normal controls (Fig. 4F). The median value of percentage of CD74^+^ cell in AML CD34^+^ cell was significantly higher than that in normal CD34^+^ cells (Fig. 4G). This result indicates that expression of surface CD74 is increased in AML CD34^+^ cells, suggesting that CD74^+^ expression is associated with LSCs of AML patients.

### 3.6 CD74 ^+^ leukemia cells exhibit an increased chemotherapy resistance and enhanced colony formation ability, and have a high percentage of cells in quiescent state

**Fig. 5:**
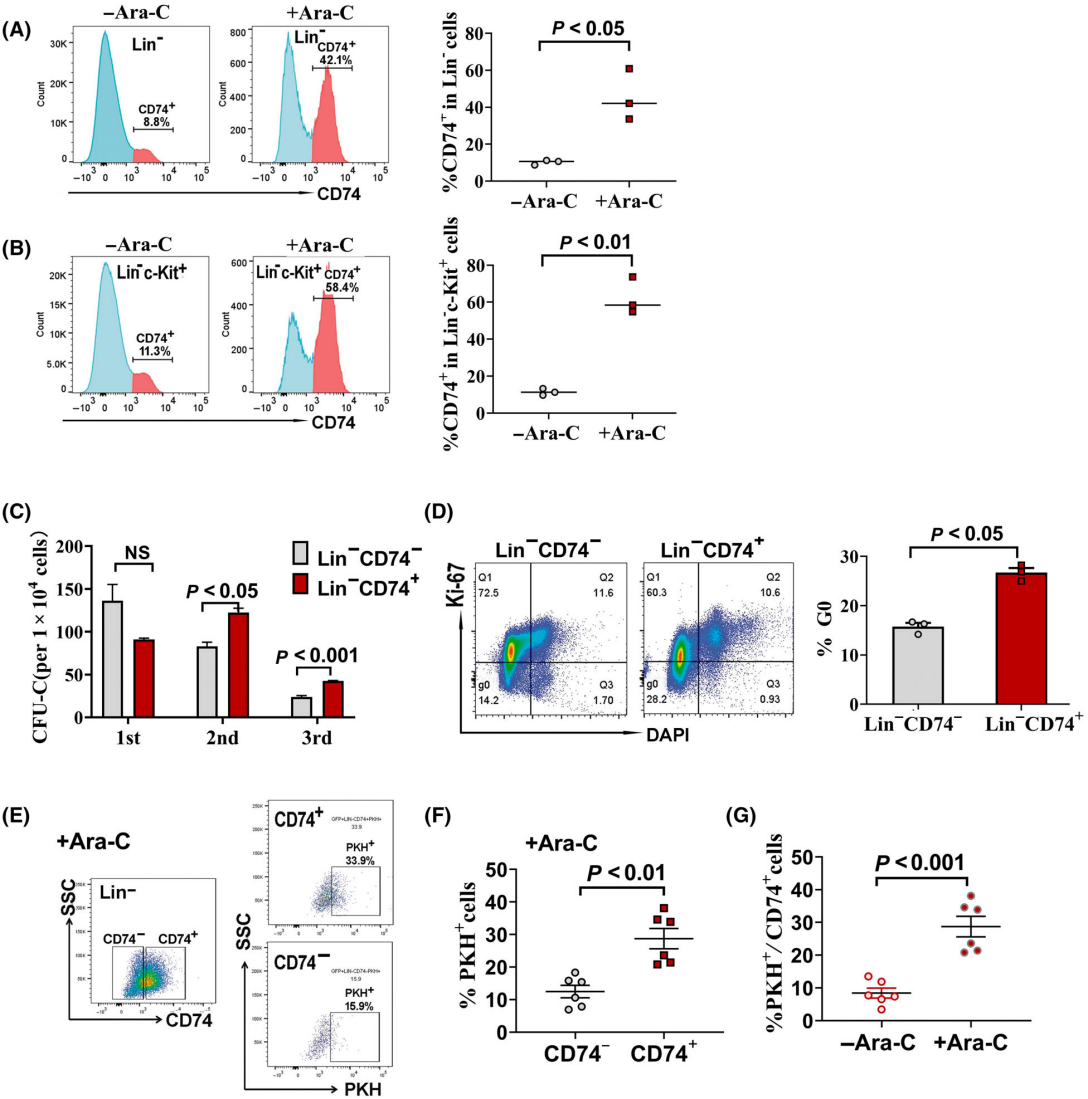
Chemotherapy resistance, colony formation ability and ratio of cells in G0 phase of CD74^+^ leukemia cells in AE9a leukemia mice. (A) Enrichment of CD74^+^ cells within the Lin^−^ leukemia cell population by Ara‐c treatment *in vivo*. Left panel: Representative flow cytometry plot of CD74^+^ cells within Lin^−^ leukemia cells from bone marrow (BM) of AE9a mice treated with or without Ara‐C. Right panel: The ratio of CD74^+^/ Lin^−^ cells from BM of AE9a mice treated with or without Ara‐C. Data are presented as mean ± SEM of three mice in one of three independent experiments. (B) Enrichment of CD74^+^ cells within the Lin^−^c‐Kit^+^ leukemia cell population by Ara‐c treatment *in vivo*. Left panel: Representative flow cytometry plot of CD74^+^ cells within Lin^−^c‐Kit^+^ leukemia cells from BM of AE9a mice treated with or without Ara‐C. Right panel: The ratio of CD74^+^/ Lin^−^c‐Kit^+^ cells from BM of AE9a mice treated with or without Ara‐C. Data are presented as mean ± SEM from three mice in one of three independent experiments. The mice received 500 mg/kg Ara‐C for 3 days beginning on day 10 after AE9a leukemia cells transplantation. CD74^+^ cell population were evaluated 1 day after the last Ara‐C treatment (A, B). (C) Colony formation ability of Lin^−^ CD74^+^ and Lin^−^CD74^−^ cells determined by serial colony formation assay. Data are presented as the mean ± SEM of three independent experiments. (D) G0 phase of the cell cycle analyzed by flow cytometry after Ki67/DAPI staining in Lin^−^CD74^+^ and Lin^−^CD74^−^ cells. One representative flow cytometry analysis is shown in the left panel. The graph on the right panel is the ratio of cells at G0 phase in Lin^−^CD74^+^ and Lin^−^CD74^−^ cell population. Data are presented as the mean ± SEM of three independent experiments. Each dot represents the value from one experiment. (E) Representative flow cytometry plot of PKH^+^ cell in CD74^+^ and CD74^−^ cells in bone marrow of Ara‐C‐treated mice transplanted with PKH26‐stained leukemia cells. The mice received 500 mg/kg Ara‐C for 3 days beginning on day 10 after the mice being transplanted with PKH26‐stained leukemia cells. CD74^+^ and PKH^+^ cell population were evaluated 1 day after the last Ara‐C treatment. (F) Percentage of PKH^+^ cell in CD74^+^ and CD74^−^ cells in bone marrow of Ara‐C‐treated mice transplanted with PKH26‐stained leukemia cell. (G) Percentage of PKH^+^ cells in CD74^+^ cells in bone marrow of Ara‐C‐treated or untreated mice transplanted with PKH26‐stained leukemia cells. Data are presented as the mean ± SEM from six mice (F, G). The statistical significances were determined using unpaired *t*‐test (A–D, F, G).

Given that CD74 is highly expressed in both mouse and human AML LSCs, we then determined whether CD74^+^ leukemia cells exhibited stemness properties. Using AE9a leukemia mice model, we first analyzed whether CD74^+^ cells could be enriched after Ara‐C treatment and represented leukemia cell population with chemotherapy resistance. In AE9a leukemia cell transplanted mice, when the GFP^+^ leukemic cells reached to 8–12% in the recipient peripheral blood at day 10 post‐transplantation, the mice received 500 mg/kg Ara‐C for 3 days. The treatment strategy is same as that shown in Fig. 3A. The percentage of CD74^+^ cell in Lin^−^ and LK cell population was determined 1 day after the last dose of Ara‐C. As shown in Fig. 5A, the proportion of CD74^+^ cells in Lin^−^ leukemia cells was significantly increased in Ara‐C‐treated mice when compared to that in untreated mice (*P* = 0.01). CD74^+^ cell population in Lin^−^ leukemia cells was enriched 4.5‐fold following Ara‐C treatment. Similarly, the proportion of CD74^+^ cells in LK leukemia cells was also significantly increased in Ara‐C‐treated mice and CD74^+^ cell population in LK leukemia cells was enriched 5.4‐fold after Ara‐C treatment (Fig. 5B). The results indicate that CD74^+^ leukemia cells are more resistant to chemotherapy.

We then performed serial colony formation assay and evaluated the self‐renewal potential of CD74^+^ leukemia cells. Lin^−^ CD74^+^ and Lin^−^ CD74^−^ leukemia cells from AE9a mice were sorted by FACS and then subjected to colony culture assay. As shown in Fig. 5C, in the first round of culture, there was no significant difference in colony numbers produced by Lin^−^ CD74^+^ and Lin^−^CD74^−^ leukemia cells. However, in the second and third rounds, Lin^−^ CD74^+^ leukemia cells produced more colonies than Lin^−^CD74^−^ cells did (*P* < 0.05 in the second round, *P* < 0.001 in the third round). This result indicates that CD74^+^ leukemia cells display high colony formation ability and implies this cell population has the potential for self‐renewal.

Furthermore, quiescence maintaining property of CD74^+^ leukemia cells, which closely associated with chemo‐resistance and self‐renewal potential, was determined. The percentages of cells in G0 phase in sorted Lin^−^ CD74^+^ and Lin^−^CD74^−^ leukemia cells were analyzed. As shown in Fig. 5D, compared to Lin^−^CD74^−^ cells, Lin^−^ CD74^+^ cells have a significantly higher percentage of cells in G0 phase (*P* < 0.05), indicating that more Lin^−^ CD74^+^ leukemia cells maintain a quiescent state.

Based on our finding that PKH^+^ leukemia cells represented slow‐replicating cells maintaining a quiescent state, we then further analyzed the percentage of PKH^+^ cells in CD74^+^ cells following Ara‐C treatment in the PKH‐labeled leukemia cells transplanted mice. As shown in Fig. 5E,F, in BM of Ara‐C‐treated mice, the percentage of PKH^+^ cells in CD74^+^ cells was significantly higher than that in CD74^−^ cells, this indicating that PKH^+^ cells were mainly enriched in chemo‐resistant CD74^+^ cell population. We also compared the ratio of PKH^+^ cells in CD74^+^ cells in Ara‐C treated or untreated mice, Fig. 5G showed that in CD74^+^ cell population, PKH^+^ cells could be highly enriched by Ara‐C treatment, indicating that CD74^+^ cells contain the majority of cells with slow‐replicating and chemo‐resistance properties. In Ara‐C untreated mice, although a lower ratio of PKH^+^ cell was found in CD74^+^ cells, it is also significantly higher than that in CD74^−^ cells (Fig. S2A,B). Lower ratio of PKH^+^ cell was found in CD74^−^ cells, however, it is also could be enriched by Ara‐C treatment (Fig. S2A,C).

Taken together, these data demonstrate that CD74^+^ leukemia cells exhibit an enhanced chemo‐resistance and self‐renewal potential, as well as quiescence property, suggesting that CD74^+^ leukemia cells represent a cell population with high stemness.

## 4 Discussion

Quiescence is critical to ensure hematopoietic stem cells (HSCs) maintenance via preventing HSCs exhaustion and protecting them from damage under stress conditions [5, 24]. In leukemic cell, cell cycle quiescence is not only important for self‐renewal potential, but also closely related to drug resistance of LSCs, which is likely responsible for leukemia relapse [4, 25]. Thereby, identification and functional characterization of quiescent leukemia cell, as well as recognition of their surface markers, will facilitate the development of therapeutic strategies to eliminate drug resistant LSCs.

Classical DNA fluorescent stains are commonly used to determine quiescent cells, however, due to the cell cytotoxicity, DNA fluorescent labeling couldn't be used for monitoring live cells that required for functional analysis. PKH labeling makes it possible to trace live cells and assess the function of label‐retaining cells subsequently [26]. In the present study, by PKH labeling of mouse AE9a leukemia cells, transplanting and monitoring the labeled cells *in vivo*, we captured a rare labeled‐retaining leukemia cell subpopulation and identified that this cell population was highly enriched with the quiescent fraction, and exhibited drug resistance and leukemia‐initiating properties.

In the analyses on the distribution of PKH intensity of transplanted PKH^+^ leukemia cells, it revealed that the proportion of PKH‐retaining cells decreased dramatically from day 1 to day 10 post‐transplantation (Fig. 1B,C), indicating that majority of leukemia cells proliferated actively. Then, PKH‐retaining cells maintained at very low levels with a percentage of about 2% until day 25. Although a stable PKH^+^ cell number was maintained, the mean fluorescence intensity (MFI) of PKH^+^ cell decreased gradually, demonstrating that PKH‐retaining cells are slow‐replicating and this slow‐replicating cell population exhibits a replicating heterogeneity. In this slow‐replicating cell population, about 37% of the cells displayed cell cycle quiescence.

In the stemness analysis, we found that in addition to drug resistance and self‐renewal properties, PKH‐retaining cells tended to be localized to the endosteal surface of BM. A similar property was reported in acute lymphoblastic leukemia cells, in which the dormant cells preferentially localized close to the endosteum, suggesting that endosteal niche may be of benefit to LSCs for their dormancy and drug resistance [23].

For GSEA on transcriptional signatures of PKH^+^ and AE9a cells in EBM, it showed that both cell populations exhibited a transcriptional signature enriched in quiescence. We also found that in quiescence gene set, the numbers of the genes with RANK METRIC SCORE > 0 were 55 in PKH^+^ vs PKH^−^ and 87 in EBM vs CBM. Venn analysis showed that 40 overlapping genes shared by these two simple pairs (Fig. S3). In addition, for the CORE ENRICHMENT genes in quiescence gene set, 26 genes contributed to PKH^+^ cells and 62 genes contributed to EBM‐located cells, and 17 CORE ENRICHMENT genes were overlapped in PKH^+^ vs PKH^−^ and EBM vs CBM. It suggests that some common quiescence‐related molecules are utilized by PKH^+^ and endosteal‐resident leukemia cells.

Based on the transcriptome profiles of PKH‐retaining and endosteal‐resident leukemia cells, *Cd74* was found to be highly expressed in both two populations. We then confirmed that cell surface CD74 was highly expressed in LSCs in mouse leukemia cell, as well as in CD34^+^ leukemia cells of AML patients. We observed that higher percentage of CD74^+^ was enriched in PKH^+^ slow‐replicating cells, in addition, PKH^+^ cells could also be enriched in CD74^+^ cell population by Ara‐C treatment. From the analyses of quiescence, colony formation and chemo‐resistance, it was shown that PKH^+^ and Lin^−^CD74^+^ cells were not exactly the same population. LSC compartment is heterogeneous [3, 27, 28], which contains heterogeneous populations of cells with variable immunophenotypes. Our results suggest that CD74 is one of the candidate surface markers within LSCs.

As a chaperone of MHC II, CD74 plays an important function in the MHC II antigen presentation pathway [29, 30]. By suppressing degradation of internalized antigens, cytoplasmic CD74 can preserve epitopes for antigen presentation and modulating antigen processing [30, 31]. For normal and malignant hematopoietic cell, in the investigation on transcriptional profiling of BM cells from AML patients and healthy donors using single‐cell RNA sequencing, Galen et al identified that CD74 was highly expressed in HSC‐like and progenitor‐like malignant cells from AML patients but was not expressed in HSCs and progenitors from normal BM [32]. Our Flow cytometry analysis data of cell surface CD74 expression in CD34^+^ cells of AML patients and normal donors supports their transcriptional data. The expression of CD74 has been reported in AML patient specimens and several AML cell lines, especially, cell surface CD74 was upregulated in AML cell lines after IFN‐γ exposure [33, 34]. In a more recent report, investigation on cell surface CD74 expression in a total of 1294 pediatric AML patients showed that the BM cells from 38% of the patients expressed CD74 and a significant number of patients with high CD74 expression had poor outcomes [35]. These reports support our finding that cell surface CD74 is highly expressed in LSCs of AML patients. In addition, they found that increased CD74 expression was associated with the pediatric AML patients with t (8;21) translocation, trisomy 8, and CEBP‐α mutations. In our AML samples, a similar result was found that high CD74 expression was observed in the patients with CEBP‐α mutation and the patients expressing AML1‐ETO fusion proteins. In mouse AML models, a moderate level of CD74 expression was found in MLL‐AF9 leukemia mice compared with that in AE9a leukemia mice (Fig. S4).

We also identified that CD74 is highly expressed in the surface of CD34^+^ AML cells. CD34^+^ AML cells represent the LSCs which retains the stemness properties including quiescence [25]. Galen's study also showed that in addition to CD74, some genes involved in self‐renewal, as well as LSC markers were also upregulated in HSC‐like and progenitor‐like AML cells [32]. In some types of solid tumors, CD74 expression was associated with high grade and poor prognosis [36, 37, 38]. Together with our finding, there is more evidence supporting that CD74 is related to stemness properties of malignant cells.

In normal condition, CD74 is expressed in MHC II–positive cells, including B cells, macrophages, and dendritic cells [39]. For hematologic malignancies, CD74 expression has been found in a variety of malignant cells of B‐cell origin, including lymphoma and multiple myeloma [17, 39, 40]. Based on the expression of CD74 in malignant cells, anti‐CD74 monoclonal antibody and its conjugates have been developed and showed antitumor activity against B‐cell neoplasms and some solid tumors [39, 41, 42, 43]. An antibody‐drug conjugates (ADC) targeting cell surface CD74 exhibits efficacy against CD74‐positive AML cells [44]. Cell surface CD74 could be rapidly internalized through the endosomal pathway, which may make CD74 an excellent ADC target for CD74^+^ AML [44, 45].

## 5 Conclusions

Conclusively, we provide evidence that slow‐cycling leukemia cell population represents LSC population with self‐renewal potential and chemo‐resistance, and CD74 is highly expressed in LSCs and associated with stemness properties of AML LSCs. Our finding suggests that CD74 may be a new candidate to serve as an immunotherapy target of AML.

## Conflict of interest

The authors declare no conflict of interest.

## Author contributions

HL, ZC, and Y Liu performed all experimental validation, analysis and interpretation of data, and wrote the manuscript. ZX analyzed the transcriptome microarray and RNA‐seq data. Y Li, HX and YX helped with establishment of mouse AML transplantable model. RG and SQ collected human samples, detected and analyzed expression of CD74 by FACS. HW analyzed the association of CD74 expression with AML subtype and the genetic subgroup. MW interpreted data and revised the manuscript. QR and JW designed and supervised the study, interpreted data, revised and approved the manuscript.

## Ethics statement

Ethics approval was obtained from Institutional Review Board of Institute of Hematology and Blood Diseases Hospital (Ref: NKRDP2021005‐EC‐2). All participants included in the study signed informed consents in accordance with the Declaration of Helsinki. The study methodologies also conformed to the standards set by the Declaration of Helsinki.

### Peer review

The peer review history for this article is available at https://www.webofscience.com/api/gateway/wos/peer‐review/10.1002/1878‐0261.13690.

## Supporting information


**Fig. S1.** Percentage of GFP^⁺^ cell in bone marrow of the mice transplanted with PKH26‐stained leukemia cell at each time point after transplantation. The presented data at each time point were obtained from two mice (n=2).

**Fig. S2.** Percentage of PHK^+^ cell in CD74⁺ and CD74^−^ cells in BM of Ara‐C‐treated or untreated mice transplanted with PKH26‐stained leukemia cells. A. Representative flow cytometry plot of PHK^+^ cell in CD74⁺ and CD74^−^ cells in BM of Ara‐C treated or untreated mice transplanted with PKH26‐stained leukemia cells. B: Percentage of PHK^+^ cell in CD74⁺ and CD74^‐^ cells in BM of Ara‐C untreated mice transplanted with PKH26‐stained leukemia cells. C. Percentage of PHK^+^ cells in CD74^‐^ cells in BM of Ara‐C treated or untreated mice transplanted with PKH26‐stained leukemia cells. Data are presented as the mean ±SEM from six mice in Ara‐C treated or untreated group (n=6). The statistical significance were determined using unpaired t‐test (B,C).

**Fig. S3.** Venn analysis of genes with RANK METRIC SCORE >0 and the CORE ENRICHMENT genes in quiescence gene set in PKH^+^ vs. PKH^−^ group and EBM vs. BM group.

**Fig. S4.** Cell surface CD74 expression in BM of AE9a and MLL‐AF9 leukemia mouse. Presented data are obtained from three mice in each group.

**Table S1.** Top genes upregulated in leukemia cell of PKH^+^ vs PKH^−^ and that located in EBM vs CBM.

## Data Availability

The supporting data related to our findings throughout our study, including the transcriptional data, will be available from the corresponding author [wangjx@ihcams.ac.cn] upon reasonable request.
