# Supplementary figures and images for: Slow‐replicating leukemia cells represent a leukemia stem cell population with high cell‐surface CD74 expression
Source: Mol Oncol. 2024 Jun 22;18(10):2554–68. doi: 10.1002/1878-0261.13690 (PMC11459046; doi:10.1002/1878-0261.13690)

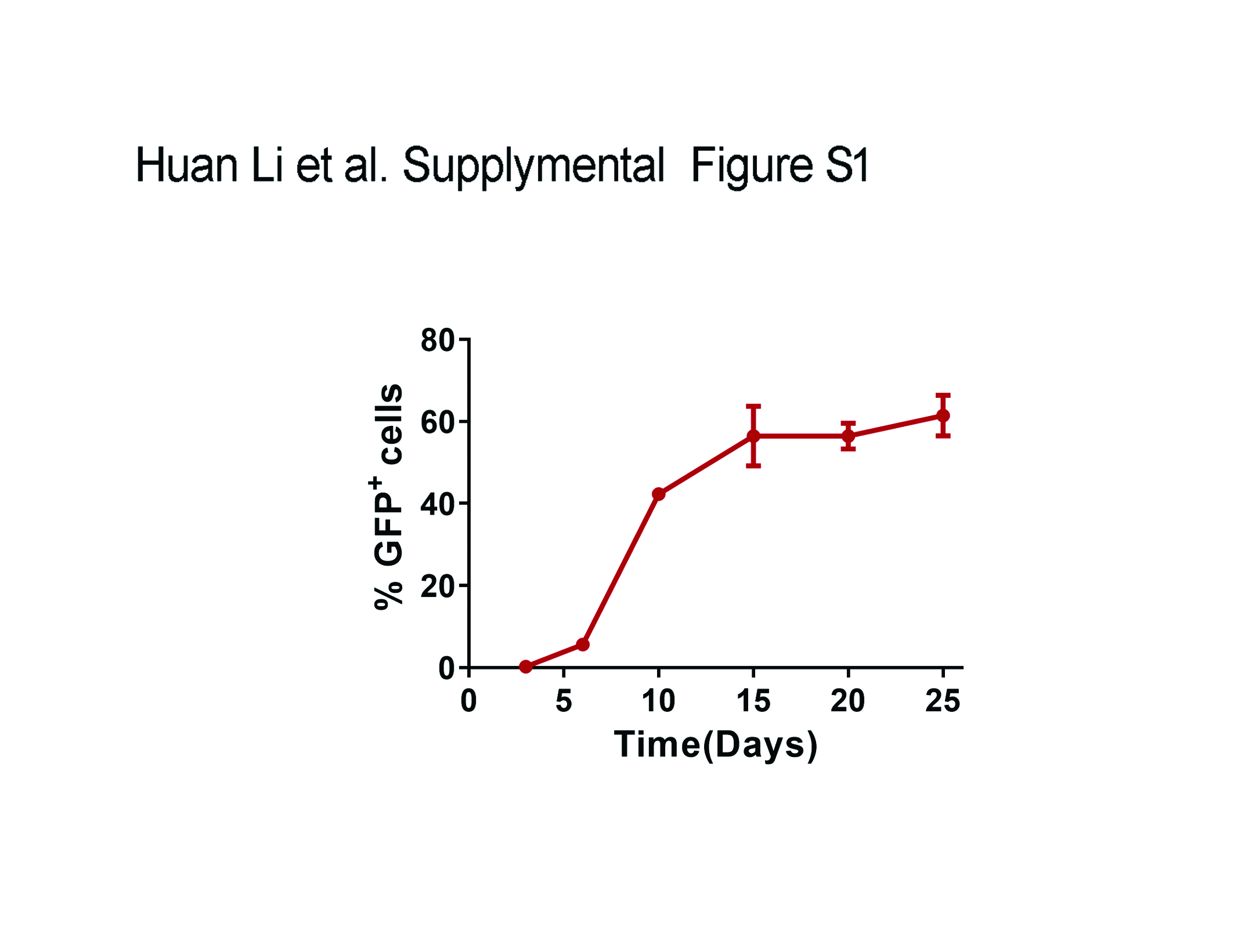

Supplement: Supplementary file 1 — Fig. S1. Percentage of GFP⁺ cell in bone marrow of the mice transplanted with PKH26‐stained leukemia cell at each time point after transplantation. The presented data at each time point were obtained from two mice (n=2). [file MOL2-18-2554-s002.tif]

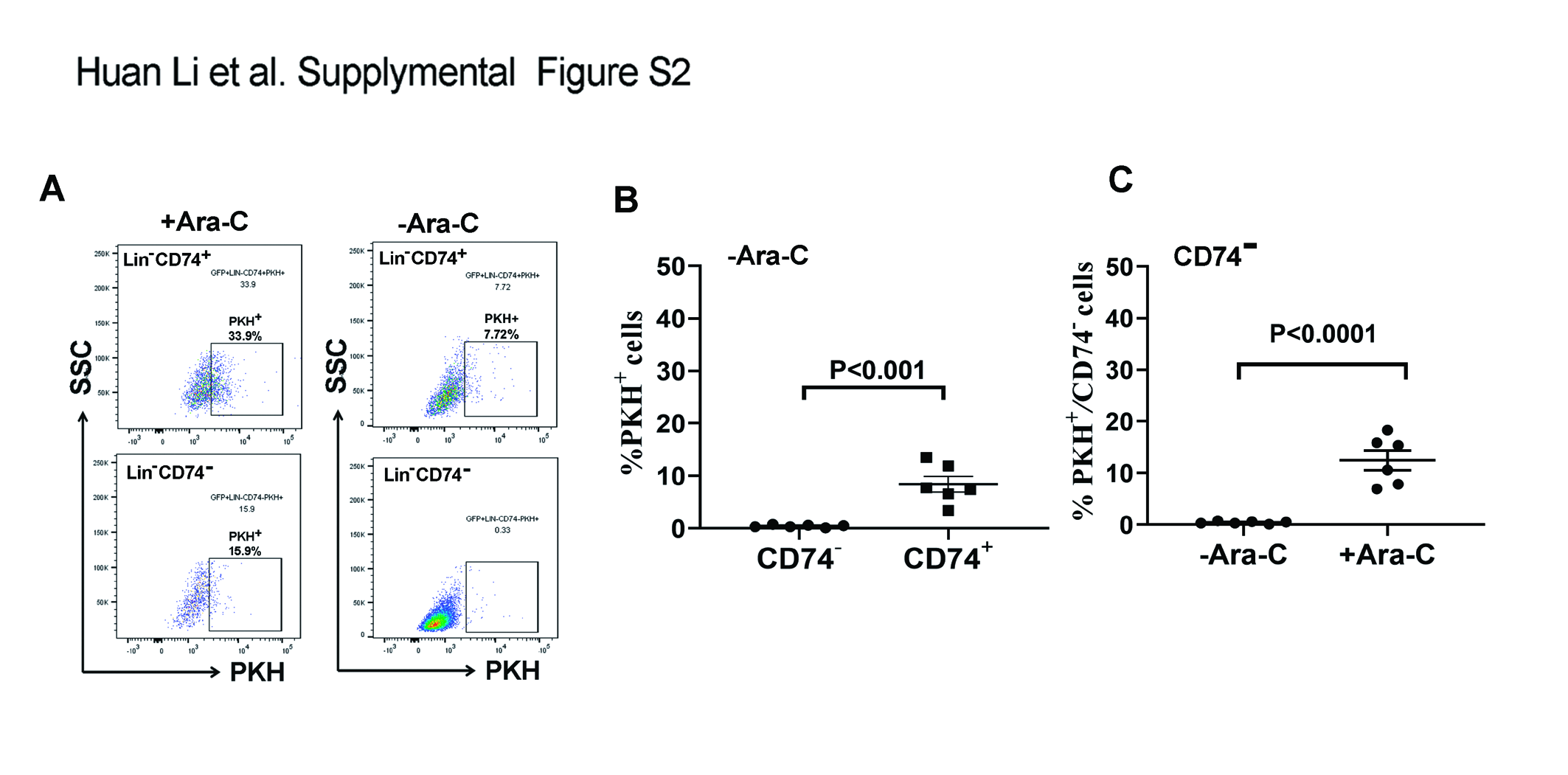

Supplement: Supplementary file 2 — Fig. S2. Percentage of PHK+ cell in CD74⁺ and CD74− cells in BM of Ara‐C‐treated or untreated mice transplanted with PKH26‐stained leukemia cells. A. Representative flow cytometry plot of PHK+ cell in CD74⁺ and CD74− cells in BM of Ara‐C treated or untreated mice transplanted with PKH26‐stained leukemia cells. B: Percentage of PHK+ cell in CD74⁺ and CD74‐ cells in BM of Ara‐C untreated mice transplanted with PKH26‐stained leukemia cells. C. Percentage of PHK+ cells in CD74‐ cells in BM of Ara‐C treated or untreated mice transplanted with PKH26‐stained leukemia cells. Data are presented as the mean ±SEM from six mice in Ara‐C treated or untreated group (n=6). The statistical significance were determined using unpaired t‐test (B,C). [file MOL2-18-2554-s004.tif]

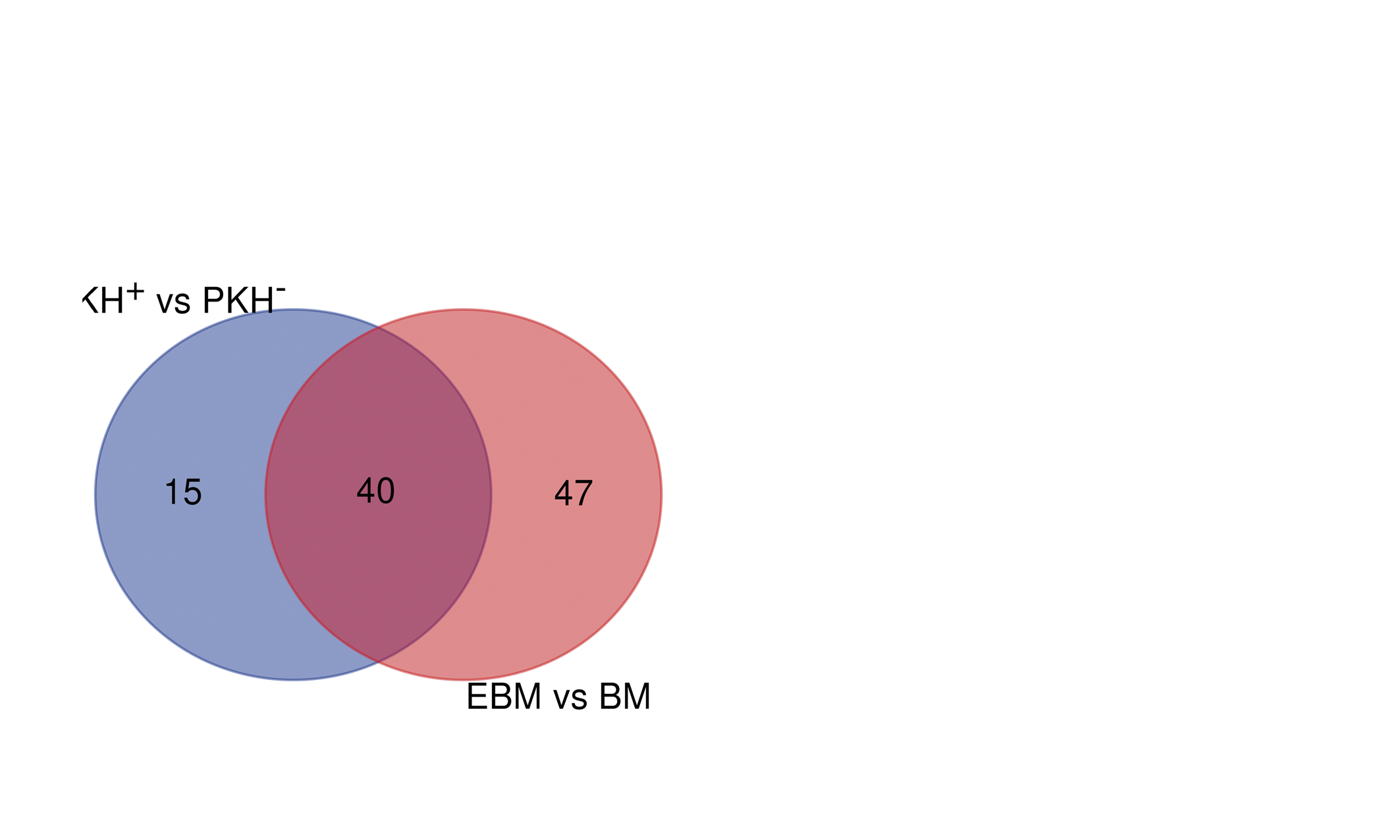

Supplement: Supplementary file 3 — Fig. S3. Venn analysis of genes with RANK METRIC SCORE >0 and the CORE ENRICHMENT genes in quiescence gene set in PKH+ vs. PKH− group and EBM vs. BM group. [file MOL2-18-2554-s005.tif]

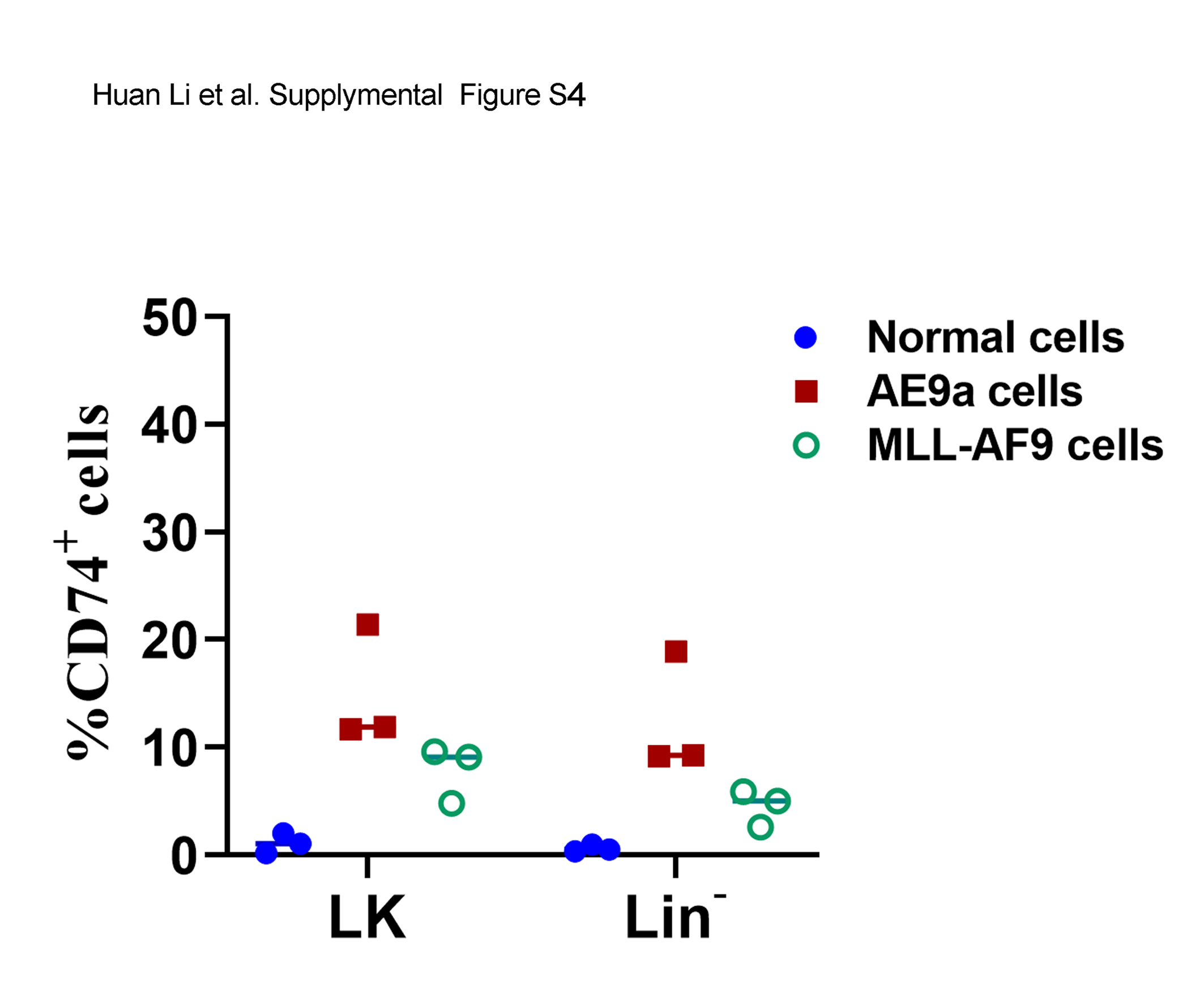

Supplement: Supplementary file 4 — Fig. S4. Cell surface CD74 expression in BM of AE9a and MLL‐AF9 leukemia mouse. Presented data are obtained from three mice in each group. [file MOL2-18-2554-s001.tif]
