# Supplementary material for: Slow‐replicating leukemia cells represent a leukemia stem cell population with high cell‐surface CD74 expression
Source: Mol Oncol. 2024 Jun 22;18(10):2554–68. doi: 10.1002/1878-0261.13690 (PMC11459046; doi:10.1002/1878-0261.13690)
Supplement: Supplementary file 5 — Table S1. Top genes upregulated in leukemia cell of PKH+ vs PKH− and that located in EBM vs CBM. [file MOL2-18-2554-s003.pdf]

Supplementary Table 1: Top genes up-regulated in leukemia cell of PKH^+ vs PKH^- and that located in EBM vs CBM

PKH^+ vs PKH^-		EBM vs CBM	
Gene. Symbol	Fold Change	Gene name	log2 Fold Change
Vcam1	11.76898724	Hspa1a	4.493235445
C1qb	9.752224459	Hspa1b	3.739108353
Fcer1g	7.724478582	Iqcn	3.475739741
Slc40a1	6.851820622	Il1b	3.249087179
Hmox1	6.352831063	H2-Ab1	2.949125654
Igkv4-53	6.14021413	Il1a	2.681102474
Lilrb4	5.907336539	Rnd1	2.660269619
Cd68	5.890761878	Bcl3	2.516102177
Emr4	5.65242051	Gm27010	2.445299781
Fcgrt	5.633220521	Trp53inp2	2.335809269
Ctsb	5.229729032	Gm29371	2.333500751
Ctsc	5.115423634	Dusp8	2.237438769
Mrc1	5.069284994	Trib1	2.126242878
Ear2	4.609441211	Cd74	2.068227992
Igsf6	4.607439446	Gm43305	2.048345748
Spic	4.538223225	Nfkbiz	2.016304918
Cfp	4.248550782		
